# Assisted HIV partner services training in three sub‐Saharan African countries: facilitators and barriers to sustainable approaches

**DOI:** 10.1002/jia2.25307

**Published:** 2019-07-19

**Authors:** Hannah Han, Serene Myers, Eveline Mboh Khan, Sarah J Masyuko, Zulmira Paredes, Francois T Chimoun, Florindo Mudender, Beatrice M Wamuti, Winifred Nambu, Emily Kemunto, Mary Mugambi, Edward Kariithi, Matthew R Golden, Pius M Tih, Thomas Welty, Carey Farquhar

**Affiliations:** ^1^ Department of Global Health University of Washington Seattle WA USA; ^2^ International Training and Education Center for Health (I‐TECH) Seattle WA USA; ^3^ AIDS Care and Prevention Program Cameroon Baptist Convention Health Services Bamenda Cameroon; ^4^ Ministry of Health National AIDS and STI Control Programme Nairobi Kenya; ^5^ International Training and Education Center for Health (I‐TECH) Maputo Mozambique; ^6^ PATH Kisumu Kenya; ^7^ Department of Epidemiology University of Washington Seattle WA USA; ^8^ Department of Medicine University of Washington Seattle WA USA; ^9^ Public Health Seattle & King County HIV/STD Program Seattle WA USA

**Keywords:** partner services, HIV/AIDS, training strategies, facilitators and barriers, sustainability, sub‐Saharan Africa

## Abstract

**Introduction:**

Healthcare worker training is essential to successful implementation of assisted partner services (aPS), which aims to improve HIV testing and linkage‐to‐care outcomes for previously unidentified HIV‐positive individuals. Cameroon, Kenya and Mozambique are three African countries that have implemented aPS programmes and are working to bring those programmes to scale. In this paper, we present and compare different aPS training strategies implemented by these three countries, and discuss facilitators and barriers associated with implementation of aPS training in sub‐Saharan Africa.

**Discussion:**

aPS training programmes in Cameroon, Kenya and Mozambique share the following components: the development of comprehensive and interactive training curricula, recruitment of qualified trainees and trainers with intimate knowledge of the community served, continuous training, and rigorous monitoring and evaluation activities. Cameroon and Kenya were able to engage various stakeholders early on, establishing multilateral coalitions that facilitated attainment of long‐term buy‐in from the local governments. Ministries of Health and various implementing partners are often included in strategic planning and delivery of training curricula to ensure sustainability of the training programmes. Kenya and Mozambique have integrated aPS training into the national HTS guidelines, which are being rolled out nationwide by the Ministries of Health and implementing partners. Continual revision of training curricula to reflect the country context, as well as ongoing monitoring and evaluation, have also been identified as key facilitators to sustain aPS training programmes. Some of the barriers to scale‐up and sustainability of aPS training include limited funding and resources for training and scale‐up and shortage of aPS providers to facilitate on‐the‐job mentorship.

**Conclusions:**

These three programmes demonstrate that aPS training can be implemented and scaled up in sub‐Saharan Africa. As countries plan for initial implementation or national scale‐up of aPS services, they will need to establish government buy‐in, expand funding sources, address the shortage of staff and resources to provide aPS and on‐the‐job mentorship, and continuously collect data to evaluate and improve aPS training plans. Development of national standards for aPS training, empowered healthcare providers, increased government commitment, and sustained funding for aPS services and training will be crucial for successful aPS implementation.

## 1 Introduction

In HIV endemic regions, improved coverage of testing services is key to achieving the first “90” of the UNAIDS 90:90:90 targets – testing at least 90% of people living with HIV by 2020 1. During the last decade, innovative testing technologies and modalities, such as voluntary counselling and testing, provider‐initiated testing and home‐based testing, have been developed to expand coverage of HIV testing services (HTS). This conscious effort has significantly increased the uptake of HIV testing. In eastern and southern Africa, people who knew their HIV status almost doubled between 2007‐2011 and 2012‐2016, and in western and central Africa the number almost quadrupled 2. It was estimated that 70% of people living with HIV knew their HIV status in 2016 2. However, a programmatic gap remains to test hard‐to‐reach populations with undiagnosed HIV infection to achieve the first “90.”

Assisted partner services (aPS), also known as index case testing or contact tracing, is a testing strategy that has been widely used for HIV and STI prevention programmes in western countries. Distinct from the disclosure counselling approach 3, where newly diagnosed HIV‐positive individuals are counselled on how to disclose and encourage their partners to test for HIV without follow‐up, aPS includes active partner notification and testing strategies with index patients. Healthcare workers offer newly diagnosed HIV‐positive clients a broad range of options and assistance in notifying and testing their sexual and drug‐injecting partners of potential HIV exposure, provide these partners testing and referral services, and link them to care if test HIV positive. aPS services targeting sexual partners has been implemented in several countries in Africa, including Cameroon, Kenya and Mozambique, to improve case finding and linkage‐to‐care for people with undiagnosed HIV infection.

aPS was first introduced in collaboration with MoH in Cameroon, Kenya, and Mozambique in 2007, 2013 and 2014, respectively, by the following partners: Cameroon Baptist Convention Health Services (CBCHS) in Cameroon, the University of Washington (UW), USA, in Kenya and International Training and Education Center for Health (I‐TECH) in collaboration with Centro de Colaboracão em Saúde (CCS) and ARIEL Foundation in Mozambique. The description of each programme is presented elsewhere 4, 5, 6, 7, 8, 9. Since the initial launch of the aPS programmes, CBCHS reached 21,000 partners between 2007 and 2015 in partnership with Elizabeth Glaser Pediatric AIDS Foundation (EGPAF), UW, and the University of North Carolina, USA. In Kenya, UW collaborated with the Kenyan MoH and other implementing partners, such as PATH, and together they reached over 24,000 partners in a 5‐year period. In Mozambique, I‐TECH conducted two pilot aPS projects in collaboration with the Mozambican MoH, CCS, and ARIEL Foundation. Subsequently, I‐TECH Mozambique was invited in 2017 to provide technical support in implementation of the national extended pilot of aPS at 26 sites supported by the U.S. Centers for Disease Control and Prevention (CDC) that did not yet include active partner notification. The aPS projects conducted in these countries showed aPS was safe, feasible and could be brought to scale in the African context 4, 5, 6, 7, 8, 9, 10. The cluster randomized controlled trial conducted in Kenya, for example, showed that the rates of partner testing, first‐time HIV testing, and testing HIV‐positive among sexual partners increased fourfold, fifteenfold and fivefold respectively in the aPS arm compared to the control arm, and no intimate partner violence (IPV) was attributable to the intervention 6.

Based in part on the success of these programmes, the World Health Organization (WHO) published new guidelines on HTS in 2016 with a strong recommendation for providing aPS as part of the comprehensive HTS package 3. Since then, many sub‐Saharan African (SSA) countries have introduced and initiated scale‐up of aPS as part of routine HIV testing and counselling services. In this paper, we present and compare different training strategies and approaches for aPS in Cameroon, Kenya and Mozambique, and discuss the facilitators and barriers to implementing aPS training in sub‐Saharan Africa.

## 2 Discussion

### 2.1 aPS training strategies

Healthcare worker training is crucial to successful implementation of aPS programmes. It plays a critical role in developing workforce capacity to provide aPS and achieve programme goals of increased partner testing, HIV case‐finding and linkage to care. Successful aPS training strategies must include effective training curricula and teaching methods, careful selection of trainees and trainers, and on‐the‐job mentorship. Refresher courses and rigorous monitoring and evaluation should also be included in the training plan to enhance healthcare worker (HCW) job performance and the quality of services provided to patients. A summary of training strategies employed by CBCHS in Cameroon, the Kenyan National Program and I‐TECH in Mozambique is shown in Table 1.

**Table 1:** Comparison of aPS training components in Cameroon, Kenya and Mozambique

	Cameroon	Kenya	Mozambique
Training Structure	Three full days in‐class training	Three to four full days in‐class training	Five full days in‐class training followed by five full days clinic‐based training
Criteria for Trainees	Staff from HIV testing entry points at health facilities, including: Psychosocial workers Laboratory technicians Chaplains Nurses	Clinical and non‐clinical providers who provide HTS and PMTCT	Community health workers, counsellors, psychologists and MCH nurses
Criteria for Trainers	Service providers with interest and extended experience in aPS activities Require: Clinical degree or MPH/PhD or Diploma in programme administration or management	Service providers with experience in HTS and trained by members of national sub‐committee on aPS as trainers of trainers Require: Clinical degree HTS/PMTCT certificate from the Kenyan Ministry of Heath National AIDS and STI Control Programme	MoH facilitators previously trained by I‐TECH in collaboration with aPS point person at I‐TECH staff office Require: Clinical degree Familiarity with aPS protocol
Delivery strategies	Didactic lectures 50% Active learning (role‐play, group discussions and activities) 50%	Didactic lectures 40% Active learning (role‐play, group discussions and activities) 60%	Didactic lectures 40% Active learning (role‐play, group discussions and activities) 60%
On‐the‐job Mentorship	• Yes	• Yes	• Yes
Monitoring and Evaluation	Daily and final evaluations Pre and post‐training competency assessment In‐facility monitoring and evaluation	Final evaluation at the end of training Pre and post‐training competency assessment In‐facility monitoring and evaluation	Daily and final evaluations Pre and post‐training competency assessment In‐facility monitoring and evaluation
Refresher Training	Occurs two months after competing initial training 2‐day	Built into overall HTS refresher training 1‐day	None
IPV Screening and Monitoring Training	Screening includes: • Definition social harms and IPV • Screening strategies for physical, verbal and sexual IPV for each identified partner • In case of IPV risk, decision‐making on alternative partner notification strategies • Index IPV referrals Monitoring of index with moderate risk of IPV includes: • Home visit one week after aPS provision • Provision of individual or couple counselling and other referral services if necessary • Additional home visits on an as‐needed basis	Screening includes: Definition social harms and IPV Screening strategies for physical, verbal and sexual IPV for each identified partner In case of IPV risk, decision‐making on alternative partner notification strategies Index IPV referrals Monitoring of index with moderate risk of IPV includes: Home visit one week after aPS provision Provision of individual or couple counselling and other referral services if necessary Additional home visits on an as‐needed basis	Screening includes: Definition social harms and IPV Screening strategies for physical, verbal and sexual IPV for each identified partner In case of IPV risk, decision‐making on alternative partner notification strategies Index IPV referrals Monitoring of index with moderate risk of IPV includes: aPS staff ask questions and collect information about IPV adverse events at every follow‐up visit after partners are disclosed to Index IPV referrals are made as appropriate

#### 2.1.1 Training curriculum

**Figure 1:**
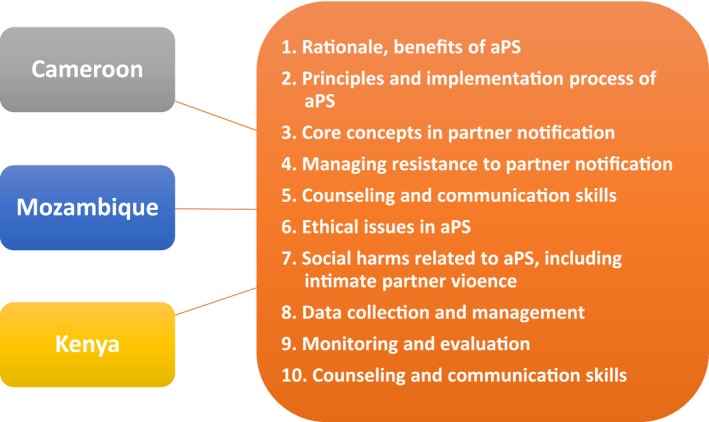
**Common topics covered in aPS training curricula in Cameroon, Kenya and Mozambique**

The training curricula deployed in Cameroon, Kenya and Mozambique cover a wide range of topics, of which the majority are common across countries (Figure 1). Key topics covered in training include the core principles of aPS—elicitation of sexual partners, partner tracing and testing, and linkage‐to‐care—counselling and communication skills, managing resistance to partner notification and screening and monitoring intimate partner violence (IPV). The training curricula in these countries highlight the importance of effective communication and building rapport in a provider‐patient relationship to address client barriers to accepting aPS, including lack of trust in HCWs and fear of IPV, breaches in confidentiality and relationship dissolution 11, 12, 13. Trainees are taught the importance of maintaining confidentiality of clients’ information and how to successfully provide patient‐centred care—critical components in improving client cooperation in aPS activities. In Cameroon, trainees take a written and oral confidentiality oath to protect the identity of the index patient under all conditions.

#### 2.1.2 Teaching approaches

A variety of teaching methods are employed in training, including didactic lectures, case studies, role‐plays, small and large group discussions, and interactive games. Role‐plays are the most popular and widely used method in aPS training in Cameroon, Kenya and Mozambique. By assuming different roles and acting out various scenarios, trainees practice new skills in a controlled environment and respond to challenging situations they might encounter in the field. This allows trainees to develop a deeper understanding of the aPS implementation process and evaluate their own progress towards the acquisition of new skills, attitudes and knowledge.

#### 2.1.3 Selection of trainees and trainers

Training for aPS primarily targets healthcare providers directly involved in HTS and HIV treatment, including HTS counsellors and community health workers. District health management teams and hospital in‐charges are often included in Kenya and Cameroon trainings to facilitate aPS integration within the routine facility processes. The Cameroon programme also recruits laboratory technicians, nurses, chaplains and social workers to participate in aPS trainings; they are also trained to perform rapid HIV testing in the field. As the first contact persons who inform clients of their HIV test results, these cadres should be familiar with aPS programme procedures, have the skills to implement aPS, and be able to make appropriate referrals for newly diagnosed HIV‐positive clients.

Trainers are carefully selected to conduct aPS trainings. When aPS was first launched in Cameroon, CBCHS used the North Carolina Department of Health training curriculum, which originated from CDC, and gradually scaled‐up aPS training in Cameroon. CBCHS staff trained HCWs from Kenya and Mozambique in 2012 and 2013 respectively incorporating field experience into the training process. Subsequently, Kenya and Mozambique implemented the first wave of training utilizing trainers from collaborators of MoH, including UW and I‐TECH. With programme expansion and government buy‐in, an increasing number of representatives from MoH and other implementing partners in Cameroon, Kenya and Mozambique are invited to co‐lead training sessions. This collaborative effort creates a sense of ownership for all parties involved. The early development of a shared vision and mission facilitates sustainable institutional capacity building efforts within MoH and other organizations to successfully implement aPS.

All three countries engage experienced HTS providers or community health workers in the trainings. Seasoned aPS staff serve as excellent resources for the new trainees; they are able to share their personal experiences conducting aPS and facilitate discussions about common challenges faced in the field. This dialogue often encourages active participation and allows trainees to learn from others’ practical experiences and lessons learned.

#### 2.1.4 On‐the‐job mentorship

Increasing emphasis is being placed on including on‐the‐job training in healthcare workforce development. Several studies in SSA have found that post‐training supportive supervision and mentorship reinforce learning process in trainees and improve their motivation and job satisfaction 14, 15, 16. This strategy was also shown to be effective in Cameroon, Kenya, and Mozambique. After completing initial training, all trainees receive ongoing on‐site support by experienced aPS providers and managers who work individually with trainees to ensure they develop the competencies necessary to provide aPS. The post‐training mentorship allows trainers to identify skills that need reinforcement; tailor the training to each trainee's particular needs; and address complex cases, barriers and unique issues that arise in each clinical setting.

Unlike Cameroon and Kenya, Mozambique employs a unique hybrid training approach where trainees participate in a 5‐day classroom‐based training followed by a clinic‐based training. The clinic‐based portion provides trainees the opportunity to shadow aPS trainers and receive feedback after practicing aPS in the clinic setting. This component of Mozambique's training programme increases self‐efficacy and job performance of newly trained HCWs.

#### 2.1.5 Monitoring and evaluation

Monitoring and evaluation are the cornerstone of successful aPS programme implementation. Successful monitoring and evaluation allow implementers to review and assess the effectiveness of training in real‐time and guide strategic planning and resource allocation. While standardized national‐level comprehensive evaluation plans for aPS training are yet to be developed, each country programme conducts regular training evaluations and in‐facility monitoring to obtain feedback and determine training outcomes.

Training evaluations cover a range of training content and assess the effectiveness of different delivery strategies. Both trainees and trainers complete the surveys at the end of training in Kenya. In Cameroon and Mozambique, daily training evaluations are also administered to tailor subsequent training sessions to better meet trainee's needs and objectives. As part of in‐class evaluation, all three countries conduct brief pre and post‐assessments to measure trainees’ attainment of learning objectives. These tests attempt to measure trainees’ knowledge of aPS, readiness for aPS programme implementation, and allow supervisors and trainers to identify participants who need additional technical support in the field.

In addition to training evaluations, Cameroon, Kenya and Mozambique conduct in‐facility monitoring and evaluation to assess on‐the‐job performance of aPS staff and provide technical assistance when needed. In Mozambique, on‐the‐job training is provided during routine follow‐up visits. Training needs are identified and addressed through mentorship in both the area of data collection and aPS counselling. Most follow‐up visits were conducted quarterly during national pilot expansion. However, the frequency of follow‐up visits varied depending on the needs of the clinics. Clinics that could benefit from additional support to improve programme implementation were identified through a monthly monitoring report system. Once identified, clinics requiring additional support were scheduled to receive more frequent visits, ranging from twice quarterly to semiannually. Barriers to implementation are also addressed at follow‐up visits to improve the staff's ability to provide aPS, and findings were shared with MoH, CDC, and other implementing partners. In Cameroon, quarterly progress review meetings provide an opportunity for experience sharing among aPS providers, capacity development through role‐playing complicated scenarios, and peer mentorship.

As part of monitoring and evaluation, all three countries collect and evaluate aPS cascade data to assess programme outcomes. The outcome indicators include the number of index clients who accepted aPS and the number of partners elicited, tested, diagnosed with HIV and linked to HIV care. Further review of cascade data within and across these three countries could allow training programmes to identify gaps in implementation and develop standardized quality improvement strategies for aPS training programmes.

#### 2.1.6 Refresher workshops

Refresher workshops are beneficial and should be offered to all trained HTS providers and community health workers at least once a year. In Cameroon, a 2‐day refresher course is held two months after the completion of initial training, whereas in Kenya, it is built into the overall HTS refresher training, which is conducted annually. Similar to the initial training, refresher workshops emphasize the importance of counselling and communication skills. Participation in refresher trainings improve HCW knowledge and skills in aPS and allow providers to stay current with the national HTS/aPS guidelines.

### 2.2 Facilitators and barriers to sustaining training strategies

**Figure 2:**
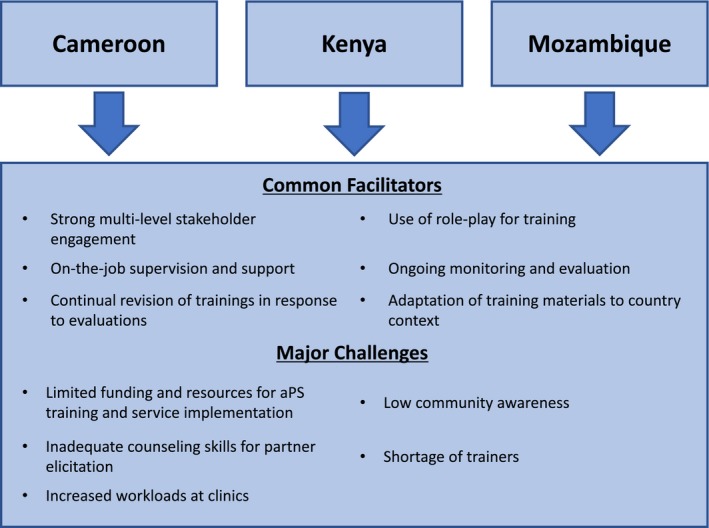
**Facilitators and barriers to implementation and scale‐up of aPS training programmes in Cameroon, Kenya and Mozambique**

There is a number of facilitators and barriers to scaling up and sustaining aPS training programmes that were identified in Cameroon, Kenya and Mozambique (Figure 2). First, ensuring sustainability of aPS training requires effective stakeholder engagement and national‐level commitment to achieve shared long‐term public health goals towards HIV epidemic control. There must be a continued collaborative effort between MoH and its partners to implement, monitor and improve aPS training. Since aPS was first introduced in Cameroon and Kenya, the countries have worked towards developing a sustainability plan to maintain and scale up aPS training nationwide. Each country involved various stakeholders early on, including regional and national health departments, with the goal of creating a strong multilevel coalition that would promote long‐term buy‐in and build support to improve testing uptake in high‐burden areas. Pilot‐testing aPS trainings before scaling‐up regionally or nationally allowed for training revision and improvement and increased stakeholder investment at scale‐up. Currently, many research groups, stakeholders and implementing partners, such as the Prevention Family AIDS Care & Education Services (FACES) and the International Center for AIDS Care and Treatment Programs (ICAP), are working together in these countries and across sub‐Saharan Africa to implement and evaluate aPS programmes.

Continuous revision of training plans and curricula to reflect the country context and evaluation results is another critical component towards sustainability of aPS training. In Cameroon, Kenya and Mozambique, the training curricula have evolved over time to reflect recommendations from training observations and evaluations to ensure successful scale‐up of aPS. A few key recommendations for improving curricula in other contexts include additional time allotted for the discussion of specific partner notification messaging, additional content related to redirecting partners when asked how their contact information was obtained; assessing IPV risk and providing appropriate referral services to clients; partner elicitation strategies to increase the number of index clients who successfully identify more than one sexual partner for aPS. In addition, Kenya is conducting qualitative analyses to assess client perspectives on aPS, which are important for evaluating effectiveness of training programmes.

Cameroon, Kenya and Mozambique have also faced several challenges to implementing aPS training. When aPS was first introduced, there was a lack of government buy‐in in Cameroon and Mozambique due to limited data that supported patient safety. After the successful pilot projects, both Cameroonian and Mozambican MoH have expressed a strong commitment to expand the aPS strategy and incorporated it into its strategic plan. However, only Kenya currently has a clear transition plan that allows complete integration of aPS training into the existing HTS and PMTCT training framework.

Limited funding and resource availability are a common barrier in all three countries. aPS trainings in these countries are currently supported by diverse international and local funders, including U.S. President's Emergency Plan for AIDS Relief (PEPFAR) and the Global Fund. This financial instability impedes development of infrastructure to regularly evaluate training outcomes and sustain aPS programmes. Centralizing funding and training activities could reduce costs, promote programme sustainability, standardize training and facilitate evaluations. Additionally, increased community engagement may help alleviate funding limitations in these countries. Currently, community awareness of aPS is low in Cameroon, Kenya and Mozambique, which poses challenges in increasing acceptability of aPS programmes in the populations served. Despite the perceived benefits, many patients show discomfort in providing information about their sexual partners for fear of breach of confidentiality, stigma and relationship conflicts 11, 17. Community sensitization of aPS through nationwide campaigns could help generate demand and public interest, which could potentially lead to increased funding and resources.

Lastly, locating trainers of trainers has been challenging in Kenya and Mozambique. In Mozambique, for example, aPS is a relatively new intervention and there are a limited number of experienced healthcare workers in aPS available to support trainings. The recent extended national pilot of aPS has relied heavily on I‐TECH staff as trainers of aPS staff in provincial clinics. Shortage of supervisors and increasing workloads at facilities has also hindered provision of effective on‐the‐job training. Facility mentors often see an average of 20 to 30 patients per day, making it challenging to allocate time to provide proper mentorship to newly trained staff. To address this concern, Kenya has begun recruiting and training community volunteers, which has shown to be highly effective in reducing workloads among HTS counsellors and supervisors. Evaluation of the cost‐effectiveness of lay healthcare workers is also ongoing in Mozambique. Additional research in this area across other sub‐Saharan contexts could have an impact in affecting policy and advocacy for increased integration of lay healthcare workers in the public sector.

In Cameroon, Kenya and Mozambique, an increasing number of public health programmes are now providing family testing alongside aPS to enhance identification and testing of previously undiagnosed HIV‐exposed children. Several programmes and studies are also underway to examine whether aPS can be used to effectively deliver HIV prevention and treatment services to key populations, including people who inject drugs (PWID). As more data become available, it will be important to evaluate programme strategies targeting these populations and their impact.

## 3 Conclusions

Scale‐up of aPS training is feasible in sub‐Saharan Africa, as seen in Cameroon, Kenya and Mozambique. As countries plan for initial implementation or national scale‐up of aPS services, they should establish government buy‐in, build the capacity of trainers, expand funding for aPS, and address the shortage of staff to provide aPS and the on‐the‐job mentorship. Development of national standards for aPS training; post‐training support such as mentorship; refresher trainings; careful selection of trainers and trainees; and continual revision of training plans and curricula to reflect recommendations from training evaluations and programme monitoring are all crucial to successful aPS workforce development.

## Competing interests

There are no conflicts of interest to declare.

## Authors’ contributions

HH wrote the commentary and led coordination of data collection and presentation. All authors contributed to the content, provided feedback and made edits as the commentary was finalized. All authors reviewed and approved the commentary.
